# Vehicle Trajectory Prediction and Collision Warning via Fusion of Multisensors and Wireless Vehicular Communications

**DOI:** 10.3390/s20010288

**Published:** 2020-01-04

**Authors:** Minjin Baek, Donggi Jeong, Dongho Choi, Sangsun Lee

**Affiliations:** Department of Electronics and Computer Engineering, Hanyang University, Seoul 04763, Korea; mjbaek@hanyang.ac.kr (M.B.); motive5aa9@hanyang.ac.kr (D.J.); cdh5375@hanyang.ac.kr (D.C.)

**Keywords:** advanced driver assistance system, trajectory prediction, risk assessment, collision warning, connected vehicles, vehicular communications, vulnerable road users

## Abstract

Driver inattention is one of the leading causes of traffic crashes worldwide. Providing the driver with an early warning prior to a potential collision can significantly reduce the fatalities and level of injuries associated with vehicle collisions. In order to monitor the vehicle surroundings and predict collisions, on-board sensors such as radar, lidar, and cameras are often used. However, the driving environment perception based on these sensors can be adversely affected by a number of factors such as weather and solar irradiance. In addition, potential dangers cannot be detected if the target is located outside the limited field-of-view of the sensors, or if the line of sight to the target is occluded. In this paper, we propose an approach for designing a vehicle collision warning system based on fusion of multisensors and wireless vehicular communications. A high-level fusion of radar, lidar, camera, and wireless vehicular communication data was performed to predict the trajectories of remote targets and generate an appropriate warning to the driver prior to a possible collision. We implemented and evaluated the proposed vehicle collision system in virtual driving environments, which consisted of a vehicle–vehicle collision scenario and a vehicle–pedestrian collision scenario.

## 1. Introduction

The incidence of road traffic crashes is one of the leading causes of death worldwide, and the reduction of the number of traffic-related crashes has become a major social and public health challenge, considering the ever-increasing number of vehicles on the road. One of the most common causes of vehicle crashes is driver inattention. One study conducted by the National Highway Traffic Safety Administration (NHTSA) reported that approximately 80 percent of vehicle crashes and 65 percent of near-crashes involved driver inattention within three seconds prior to the incident [1]. Taking into account that human life expectancy is continuously getting longer, it has become crucial that we assist those who are older and those who are physically impaired in driving and achieve higher road safety measures through research and development of advanced driver assistance systems (ADAS) technology.

The safety functions of ADAS require accurate information on the environment surrounding the vehicle. A popular approach in recent years to obtain the information on the vehicle surroundings involves fusing the data generated by multiple types of sensors (e.g., radar, lidar, and cameras) equipped on the vehicle [2,3,4,5,6,7]. This way, it is possible to overcome the functional and environmental limitations of each type of sensor and generate the estimate of the state of each surrounding object with higher accuracy. However, this sensor fusion approach has its limits on the reliability and data collection range. The sensor accuracy of driving environment information is affected by a number of factors such as weather and solar irradiance. In addition, no data can be acquired when the target is outside the field of view of the sensors or when the line of sight to the target is obstructed. In order to further enhance road safety, it is therefore critical to improve the reliability and the detection range of the perception system and also find a way to obtain information on objects in non-line-of-sight (NLOS) regions.

A wireless vehicular communication system can be viewed as a new type of automotive sensor that allows engineers to design the next generation of ADAS, enabling drivers to exchange information on their own vehicles as well as the environment surrounding them. Whereas on-board sensor data obtained with radar, lidar, and cameras enable the estimation of target vehicle information such as relative position, speed, and heading, vehicular communication data additionally provide us with the best possible measurements on vital vehicle data including speed, yaw rate, and steering angle, which are obtained directly from the remote vehicle bus. This communication network can further extend its reach when vehicles, roadside infrastructures, and vulnerable road users (e.g., pedestrians, cyclists, and motorcyclists) are equipped with wireless communication devices. Wireless vehicular communications, often referred to as vehicle-to-everything (V2X) communications, can be classified into different types including vehicle-to-vehicle (V2V), vehicle-to-infrastructure (V2I), and vehicle-to-pedestrian (V2P) communications. While V2V communications involves two or more vehicles exchanging data with each other, V2I communications allows data exchange between vehicles and roadside units. Furthermore, V2P communications involves vehicles exchanging data with pedestrians. Studies have shown that combining V2V and V2I technologies can help address about 80 percent of all vehicle crashes [8].

**Figure 1:**
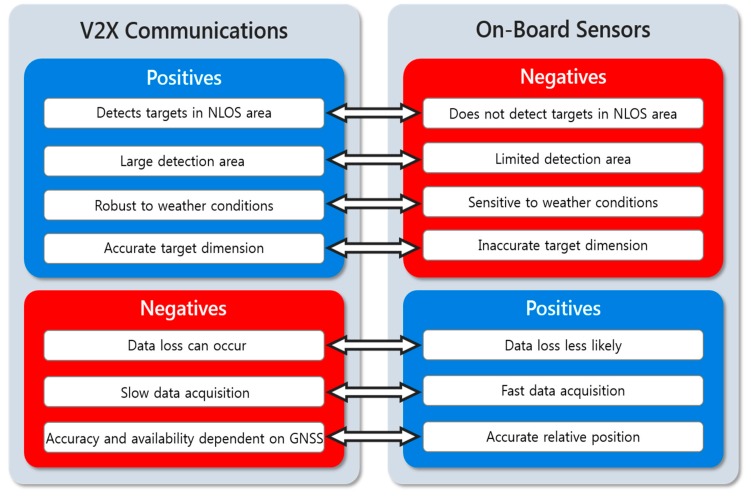
Positive and negative characteristics of perception using vehicle-to-everything (V2X) communications and on-board automotive sensors for remote sensing.

Such significant advantages of V2X communications in road safety can become even more augmented when combined with the on-board sensor measurements via data fusion. Figure 1 summarizes the positives and the negatives of perception through V2X communications and those of remote sensing with on-board sensors such as radar, lidar, and cameras. The two groups of data complement each other, resulting in a more accurate, robust, and complete perception of the vehicle surroundings. As mentioned earlier, the implementation of V2X communications greatly enhances the perception capability, as it enables detection of targets in NLOS regions and extends the detection range up to 1 km [9], while the longest detection range that can be achieved with on-board sensors is 200–250 m (through radar systems). Exchanging V2X communication data is possible regardless of weather conditions, whereas the accuracy and reliability of on-board sensors can be significantly reduced by adverse weather conditions such as rain, snow, and fog [10]. Furthermore, safety applications of camera systems such as collision warning and pedestrian detection are often inactive in a dark environment or during night time. V2X communication data also include accurate target dimension information (width, length, and height), but the dimension information obtained with on-board sensors are often inaccurate or even unavailable due to the effects of occlusion and the limitations of the sensor field of view (FOV). On the other hand, there are some negative aspects to perception based solely on V2X communications. Transmitted V2X communication data can be delayed or even lost in an adverse radio frequency propagation environment (e.g., blockage and multipath) and/or a high communication channel load scenario (e.g., heavily congested urban intersections). In addition, V2X safety messages such as the basic safety message (BSM) are transmitted at a period of 100 ms, whereas on-board sensor measurements can be collected with a period of about 50 ms or even at a faster rate depending on the sensor model. Locating targets through V2X communications is also limited in that vehicles must be equipped with vehicular communication devices to participate in the exchange of the safety messages, and that the accuracy and reliability of positioning are largely dependent on the quality and availability of the global navigation satellite system (GNSS) signals. In an environment where GNSS signals are not available (e.g., inside a tunnel and under an overpass), vehicles can no longer transmit the safety messages, which results in a discontinuous acquisition of data on surrounding vehicles.

In this paper, we propose a method for vehicle trajectory prediction and collision warning through fusion of multisensors and V2X communications. In order to enhance the perception capabilities and reliability of traditional on-board sensors, we employ a Kalman filter-based approach for a high-level fusion of radar, lidar, camera, and V2X communication data. To verify the performance of the proposed method, we constructed co-simulation environments using MATLAB/Simulink and PreScan [11], which is designed for simulation of ADAS and active safety systems. In addition to radar, lidar, and camera sensor systems, the host vehicle is equipped with a dedicated short-range communications (DSRC) transceiver, which enables the collection of information on the surrounding vehicles and vulnerable road users (VRUs) equipped with DSRC devices through exchanging safety messages. The performance of the proposed vehicle collision warning system is evaluated in a vehicle–vehicle collision scenario and a vehicle–pedestrian collision scenario.

The rest of the paper is organized as follows. Section 2 introduces related research work. In Section 3, we describe the architecture of the proposed system and discuss background information about automotive sensors for remote sensing and V2X communications. The proposed method for vehicle collision warning is presented in Section 4, and the experimental results are given in Section 5. Finally, Section 6 concludes the paper by summarizing the main points and addressing future work.

## 2. Related Work

Vehicle collision warning systems have been studied by many researchers. Typical vehicle collision warning systems are based on sensor measurements from radar and camera sensors. Vehicle collision warning and automatic partial braking systems based on radar sensors that have been implemented in commercially available Mercedes-Benz cars are described in [12]. A vehicle collision warning system with a single Mobileye camera is presented in [13], where rear-end collision scenarios are considered and the warning is generated based on the time-to-collision (TTC) calculation. More recently, there have been efforts to develop cooperative collision warning systems that utilize vehicular communications. In [14], a crossroad scenario with two vehicles equipped with GPS receivers and vehicular communication devices is considered, where the trajectory prediction is performed with a Kalman filter and TTC is used for the collision risk indicator. A rear-end collision warning model based on a neural network approach is presented in [15], where participating vehicles are equipped with GPS receivers and vehicular communication devices and are assumed to be moving in the same lane.

Despite the advantages of vehicular communications, the cooperative sensing approach based on vehicular communications and on-board sensor fusion has not been examined extensively yet by researchers. Inter-vehicle object association using point matching algorithms is proposed in [16] to determine the relative position and orientation offsets between measurements taken by different vehicles. In [17], a vision-based multiobject tracking system is presented to check the plausibility of the data received via V2V communications. Radar and V2V communication fusion approach is suggested in [18] for a longer perception range and lower position and velocity errors. In the case of maritime navigation, the automatic radar plotting aids (ARPA) and the automatic identification system (AIS) technologies are widely implemented to identify and track vessels and to prevent collisions between vessels based on radar measurements as well as static and dynamic information (e.g., vessel name, call sign, position, course, and speed) of other AIS-equipped vessels exchanged over the marine VHF radio channels [19,20]. Although these papers present promising applications, the potential of the fusion of on-board sensor data and V2X communication data in the context of ADAS applications, such as vehicle collision prevention, has not been extensively investigated.

## 3. System Overview

As each type of sensors has its advantages and disadvantages, combining data from multiple types of sensors is necessary in order to maximize detection and tracking capability. In this work, a high-level fusion of radar, lidar, cameras, and V2X communication data was performed to predict the trajectories of the nearby targets and generate an appropriate warning to the driver prior to a possible collision. In an effort to perform simulations under close-to-real conditions, the characteristics of local environment perception sensors that have been widely considered for ADAS functions in commercially available vehicles were employed.

### 3.1. Architecture of the Proposed System

**Figure 2:**
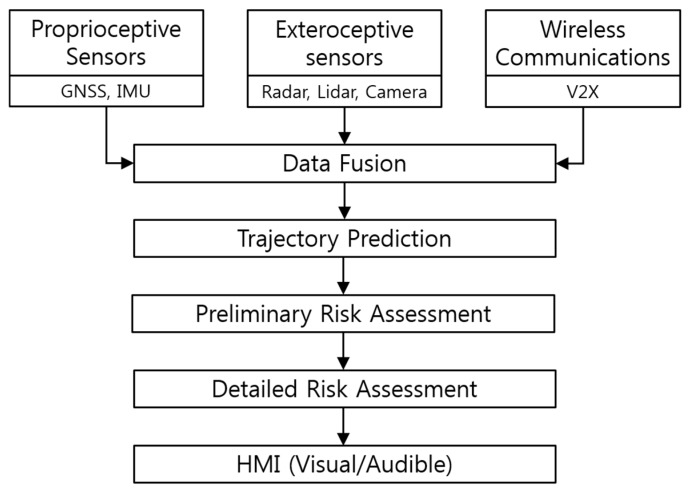
Block diagram summarizing the steps for collision warning generation.

The framework of the proposed vehicle collision warning system is illustrated in Figure 2. The first step of the proposed system involves perception. For the purpose of estimating the relative position of the target in the surrounding space with respect to the host vehicle, the host vehicle obtains the relative range and azimuth from the radar and the lidar, the relative lateral and longitudinal position from the camera, and the GNSS measurements of the remote target as well as its dynamic information such as speed and yaw rate via the DSRC transceiver. The measurements from each sensor are processed with a Kalman filter algorithm, which reduces the measurement noise and outputs the state and error covariance at each time step. Note that, in the case of computing the relative target position and orientation from V2X communication data, it is necessary to consider the heading and GNSS measurements of the host vehicle as well. A high-level fusion is performed using the estimated quality scores for sensor data, which are based on the error covariance computed through the prediction and update steps of the Kalman filter. Trajectory prediction for the targets detected in the perception stage is performed by employing the constant turn rate and velocity (CTRV) motion model. In risk assessment steps, possible vehicle collisions are detected based on the results from the previous trajectory prediction step. A preliminary assessment that requires significantly less computation load is first carried out to detect possible collisions, and if collisions are expected, a more detailed assessment is performed to estimate precise TTC. Finally, appropriate visual and audible warnings are generated to the driver based on the TTC estimate, where the warning information is provided through the human–machine interface (HMI) in four different threat levels.

### 3.2. Automotive Sensors for Remote Sensing

We selected on-board sensors that have already been adopted in production vehicles such that by adding V2X communication devices we can evaluate the benefits of introducing V2X communications to today’s vehicles in terms of road safety. The types of sensors installed on vehicles produced in recent years include radar, cameras, and also lidar, which enable ADAS features such as forward collision warning (FCW), automatic emergency braking (AEB), adaptive cruise control (ACC), and lane keeping assist system (LKAS).

Automotive radar, which is an active ranging sensor designed for detecting and tracking remote targets in the surrounding environment, is one of the most used ranging sensors for ADAS functions these days. The most widely found long-range radar sensors on production vehicles include Delphi ESR, Bosch LRR, and Continental ARS series, of which characteristics are shown in Table 1. The specification values are from the respective manufacturer’s specification sheet. In this work, the technical data of Delphi ESR were employed to model the radar in the experimental environment.

Lidar is an active ranging sensor that operates in a similar fashion to radar except that it utilizes light rather than radio waves. Most automotive lidars currently use near-infrared light with a wavelength of 905 nm. Lidar became a popular choice for automated driving technology research since it was used by a large number of teams who participated in the DARPA Grand Challenges. Lidar offers more accurate ranging performance compared with radar and cameras, but despite its advantage, most automakers are yet to adopt lidar mainly due to its tremendous cost. However, it appears that automakers will gradually consider using lidar in the near future because low-cost lidar sensors are becoming more available. Audi became the first automaker to adopt lidar in the production vehicle when they recently started shipping their flagship sedan equipped with an on-board lidar sensor [21]. The performance of the Ibeo Scala sensor is summarized in Table 2.

Contrary to other ranging sensors, vision sensors do not directly provide range information. Instead, range information is often estimated using the road geometry and the point of contact of the vehicle and the road [22], optical flow velocity vectors [23], bird’s-eye view [24], and object knowledge [24]. Considering that the detection and tracking performance of a vision-based system may largely vary depending on the algorithm used, the technical data of the Mobileye vehicle detection system, as reported in [22], were employed to model the vision sensor. Table 3 shows the performance characteristics of the Mobileye system.

### 3.3. V2X Communications

The IEEE 802.11p and the IEEE 1609 family of standards are collectively called wireless access in vehicular environments (WAVE) standards. The IEEE has developed the IEEE 802.11p as an amendment to the IEEE 802.11 to include vehicular environments [25]. This amendment was required to support wireless communications among vehicles and infrastructure. The IEEE 1609 protocol suite is a higher-layer standard based on the IEEE 802.11p. In the case of V2V communications, on-board units (OBUs) are installed in vehicles to enable wireless communication. These devices operate independently and exchange data using the 5.9 GHz DSRC frequency band, which is divided into seven 10-MHz channels. One of them is the control channel (CCH), which is used for safety and control messages, while other six are the service channels (SSHs), which are used for data transfer [26]. The characteristics of the WAVE standards are summarized in Table 4.

For the purpose of V2X communications, the host vehicle in this work is equipped with a DSRC antenna in addition to the sensors described in the previous section. This makes it possible for the host vehicle to gather information on the remote vehicles in the surrounding area (up to a distance of 1000 m) by exchanging BSMs, which are sent over the CCH channel with a period of 100 ms. The BSM, which is defined in the SAE J2735 message set dictionary [27], contains safety data regarding the vehicle state such as the GNSS position, speed, heading, and yaw rate of the vehicle, as well as the vehicle size. A BSM consists of two parts: Part I and Part II. The BSM Part I contains the core data that must be included in every BSM, whereas the BSM Part II content is optional. Table 5 describes the data contained in a BSM.

Similar to the BSM, the personal safety message (PSM) contains important kinematic state information on VRUs, such as pedestrians, bicyclists, and road workers. It is possible to detect VRUs located within the DSRC coverage area by collecting the PSMs transmitted from the VRU communication devices. The PSM, which is also defined in the SAE J2735 message set dictionary [27], is currently under development, but the core data elements that must be included in a PSM are specified in advance, as shown in Table 6.

The accuracy of the BSM and the PSM information we assumed in the implementation of the proposed vehicle collision system is presented in Table 7. For the BSM, typical measurement noise characteristics of a relatively simple differential GPS (DGPS) receiver, as well as those of a wheel speed sensor and a yaw rate sensor are considered. It is important that the position data included in the BSM meet a lane-level accuracy, which is described in the United States Department of Transportation (USDOT) report on vehicular safety communications [28] as a minimum relative positioning requirement for collision warning applications. With regard to the PSM, the parameter settings for the VRU safety as reported in the SAE J2945/9 VRU safety message performance requirements [29] are employed in this work for V2P communications.

## 4. Implementation

### 4.1. Kalman Filtering

A Kalman filter-based approach was employed in this work for high-level fusion of V2X communications and on-board automotive sensors for remote sensing. Kalman filtering [30,31,32] is a recursive algorithm that keeps track of the state estimate as well as the uncertainty of the estimate, given the prior knowledge of the state and the measurements collected at the present time. Kalman filtering enables to reduce the measurement noise and obtain the errors associated with each estimated state element. In order to detect the current locations of the remote targets and predict their future trajectories, we utilized position, speed, heading, yaw rate, and size information from V2X communications; range and azimuth information from both radar and lidar; and relative longitudinal and lateral distance information from the camera. In addition, the position and heading measurements from the host vehicle were used to compute the relative position and heading to the target with respect to the host vehicle.

The motion equations of remote targets are typically presented in Cartesian coordinates. However, automotive ranging sensors such as radar and lidar provide measurements in polar coordinates, so transformation to Cartesian coordinates is necessary. Polar-to-Cartesian transformation is a nonlinear process, for which an extended Kalman filter (EKF) is often used. EKF is obtained via a linear approximation of a nonlinear system, and this is consistent only for small errors [33]. A converted measurement Kalman filter performs the coordinate transformation without bias and computes the correct covariance for the converted measurements. This filter is nearly optimal and achieves higher accuracy compared with EKF [34]. The unbiased converted measurement Kalman filter algorithm as presented in [31,35] was employed in this work.

The state vector at time step k is defined by

(1) $$x_{k}={\left[X_{k}\;\;Y_{k}\;\;v_{x,k}\;\;v_{y,k}\right]}^{T}$$

where $X_{k}$ and $Y_{k}$ describe the position of the target, and $v_{x,k}$ and $v_{y,k}$ describe the target relative velocity in longitudinal and lateral directions, respectively. The measured range and azimuth are

(2) $$r_{m}=r+\omega_{r}$$

(3) $$\theta_{m}=\theta +\omega_{\theta }$$

where r and θ are the true range and azimuth values. The range and azimuth measurement noises are denoted by $\omega_{r}$ and $\omega_{\theta }$, respectively, of which error standard deviations are $\sigma_{r}$ and $\sigma_{\theta }$. The unbiased converted measurements are

(4) $$x_{m}=b_{1}^{-1}r_{m}\cos\theta_{m}$$

(5) $$y_{m}=b_{1}^{-1}r_{m}\sin\theta_{m}$$

where $b_{1}=E\left[\cos\omega_{\theta }\right]=e^{-\sigma_{\theta }^{2}/2}$. The unbiased converted measurement vector $z_{k}$ is

(6) $$z_{k}={\left[x_{m}\;\;y_{m}\right]}^{T}$$

and the state ${\hat{x}}_{k|k-1}$ and error covariance ${\hat{P}}_{k|k-1}$ are predicted from time step k−1 to time step k by

(7) $${\hat{x}}_{k|k-1}=A{\hat{x}}_{k-1|k-1}$$

(8) $${\hat{P}}_{k|k-1}=A{\hat{P}}_{k-1|k-1}A^{T}$$

where the state transition matrix A is defined as

(9) $$\;A=\left[\;\begin{array}{cc} \begin{array}{c} 1\\ 0\\ \begin{array}{c} 0\\ 0 \end{array} \end{array} & \;\;\begin{array}{cc} \begin{array}{c} \begin{array}{c} 0\\ 1 \end{array}\\ 0\\ 0 \end{array} & \;\begin{array}{cc} \begin{array}{c} \Delta t\\ 0\\ \begin{array}{c} 1\\ 0 \end{array} \end{array} & \begin{array}{c} \begin{array}{c} 0\\ \Delta t \end{array}\\ 0\\ 1 \end{array}\; \end{array} \end{array} \end{array}\right].$$

The elements of the measurement error covariance $R_{k}$ obtained from the unbiased conversion are given by

(10) $$R_{11,\;k}=\mathrm{var}(x_{m})=\left(b_{1}^{-2}-2\right)r_{m}^{2}\cos^{2}\theta_{m}+\frac{1}{2}\left(r_{m}^{2}+\sigma_{r}^{2}\right)\left(1+b_{2}\cos2\theta_{m}\right)$$

(11) $$R_{22,\;k}=\mathrm{var}(y_{m})=\left(b_{1}^{-2}-2\right)r_{m}^{2}\sin^{2}\theta_{m}+\frac{1}{2}\left(r_{m}^{2}+\sigma_{r}^{2}\right)\left(1-b_{2}\cos2\theta_{m}\right)$$

(12) $$R_{12,\;k}=\mathrm{cov}(x_{m},y_{m})=(\frac{1}{2}b_{1}^{-2}r_{m}^{2}+\frac{1}{2}\left(r_{m}^{2}+\sigma_{r}^{2}\right)b_{2}-r_{m}^{2})\sin2\theta_{m}$$

where $b_{2}=E\left[\cos2\omega_{\theta }\right]=e^{-2\sigma_{\theta }^{2}}$. Prior to updating the state and the error covariance, the Kalman gain $K_{k}$ is computed by

(13) $$K_{k}={\hat{P}}_{k|k-1}H^{T}\left(H{\hat{P}}_{k|k-1}H^{T}+R_{k}\right)$$

where the measurement function matrix H is defined as

(14) $$H=\left[\;\begin{array}{cc} 1 & 0\\ 0 & 1 \end{array}\;\;\;\;\;\begin{array}{cc} 0 & 0\\ 0 & 0 \end{array}\;\right].$$

Then the state ${\hat{x}}_{k|k}$ and the error covariance ${\hat{P}}_{k|k}$ are updated as

(15) $${\hat{x}}_{k|k}={\hat{x}}_{k|k-1}+K_{k}\left(z_{k}-H{\hat{x}}_{k|k-1}\right)$$

(16) $${\hat{P}}_{k|k}=(I-K_{k}H){\hat{P}}_{k|k-1}.$$

For filtering data from the vision sensor and V2X communications, we utilized a linear Kalman filter [30,31,32] because a polar-to-Cartesian conversion was not necessary for the data we obtained from the two sources. A linear Kalman filter is similar to the filtering process described above, but without the steps for the unbiased conversion. For the purpose of combining the filtered information from multiple sources, we estimated their quality scores based on the error covariance matrices. The quality score matrix $W_{j,\;k|k}$ at time step k for the state obtained from the jth source is given by

(17) $$W_{j,k|k}={\left[\;\overset{n}{\underset{i=1}{\sum }}{\hat{P}}_{i,k|k}{}^{-1}\;\right]}^{-1}{\hat{P}}_{j,k|k}{}^{-1}$$

(18) $$\overset{n}{\underset{j=1}{\sum }}W_{j,k|k}=I$$

where ${\hat{P}}_{j,k|k}$ is the updated error covariance for the jth sensor and I is an identity matrix. Finally, the weight average state ${\bar{x}}_{k|k}$ for time step k is

(19) $${\bar{x}}_{k|k}=\overset{n}{\underset{j=1}{\sum }}W_{j,k|k}\;{\hat{x}}_{j,k|k}$$

where ${\hat{x}}_{j,k|k}$ is the updated state based on the information collected from the jth source.

### 4.2. Trajectory Prediction and Risk Assessment

Trajectory prediction for each detected remote target is performed by employing a CTRV model. The CTRV state space is constructed with the fused target state estimate as well as the heading and yaw rate information, which was obtained with V2X communications and then filtered with a Kalman filter. Note that the yaw rate of the target was set to zero if the safety message was transmitted from a VRU, considering that yaw rate is not included in the PSM core data. The CTRV state space at time step k is defined as

(20) $$x_{k}={\left[X_{k}\;\;Y_{k}\;\;v_{k}\;\;\vartheta_{k}\;\;\omega_{k}\right]}^{T}$$

where $X_{k}$ and $Y_{k}$ describe the relative distance to the target in longitudinal and lateral directions, respectively; $v_{k}$ is the target velocity; $\vartheta_{k}$ is the relative heading of the target; and $\omega_{k}$ is the target yaw rate. The state transition equation for calculating the state at time step k+1 can be written as

(21) $$x_{k+1}=x_{k}+\left[\begin{array}{c} \begin{array}{c} \frac{v_{k}}{\omega_{k}}\left(\sin\left(\vartheta_{k}+\omega_{k}\Delta t\right)-\sin\left(\vartheta_{k}\right)\right)\\ \;\frac{v_{k}}{\omega_{k}}\left(-\cos\left(\vartheta_{k}+\omega_{k}\Delta t\right)+\cos\left(\vartheta_{k}\right)\right)\\ 0 \end{array}\\ \omega_{k}\Delta t\\ 0 \end{array}\;\right]\;.$$

**Figure 3:**
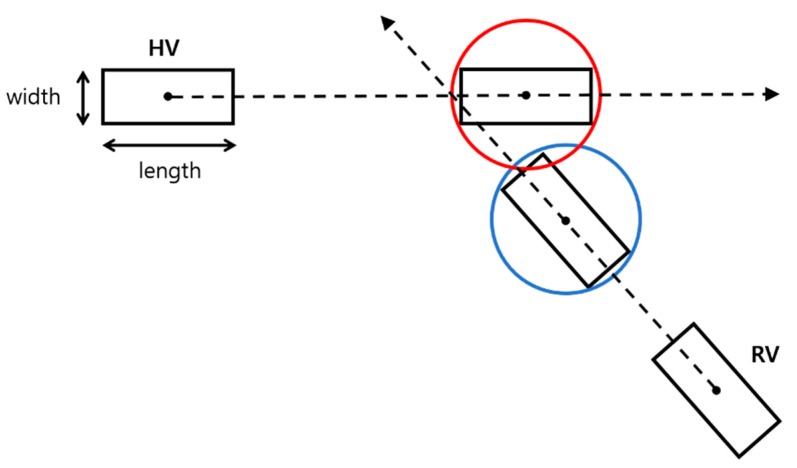
Illustration for finding a possible collision event using the predicted trajectories of the host vehicle and the remote vehicle.

The estimated trajectory of each remote target is then compared with the estimated trajectory of the host vehicle in order to determine whether or not the host vehicle will collide with the remote target. The possibility of a collision is determined by applying a circle model as shown in Figure 3, which illustrates an example for a vehicle–vehicle collision.

The radius of the host vehicle $R_{HV}$ and the radius of the remote vehicle $R_{RV}$ are defined as

(22) $$R_{HV}=\frac{\sqrt{W_{HV}{}^{2}+L_{HV}{}^{2}}}{2}$$

(23) $$R_{RV}=\frac{\sqrt{W_{RV}{}^{2}+L_{RV}{}^{2}}}{2}$$

where $W_{HV}$ and $L_{HV}$ are the width and the length of the host vehicle; and $W_{RV}$ and $L_{RV}$ are the width and length of the remote vehicle. A possible collision is detected if the inequality

(24) $$\sqrt{{\left(X_{HV}-X_{RV}\right)}^{2}}+\sqrt{{\left(Y_{HV}-Y_{RV}\right)}^{2}}\leq R_{HV}+R_{RV}$$

is true. In the case of finding a vehicle–pedestrian collision, the size of the bounding box of a VRU was set according to the dimensions stated in the European New Car Assessment Programme (Euro NCAP) test protocol for AEB VRU systems [36], which are 0.5 m and 0.6 m for an adult pedestrian and 0.5 m and 0.711 m for a child pedestrian.

**Figure 4:**
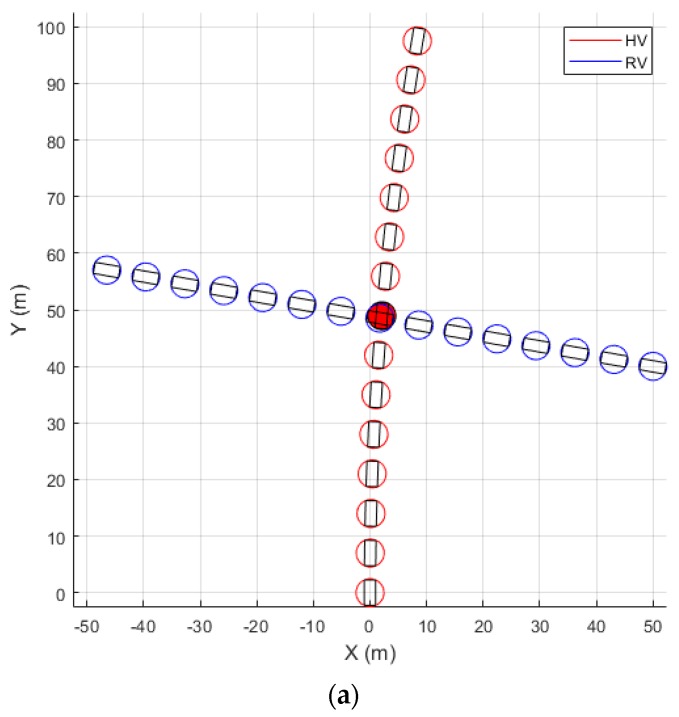

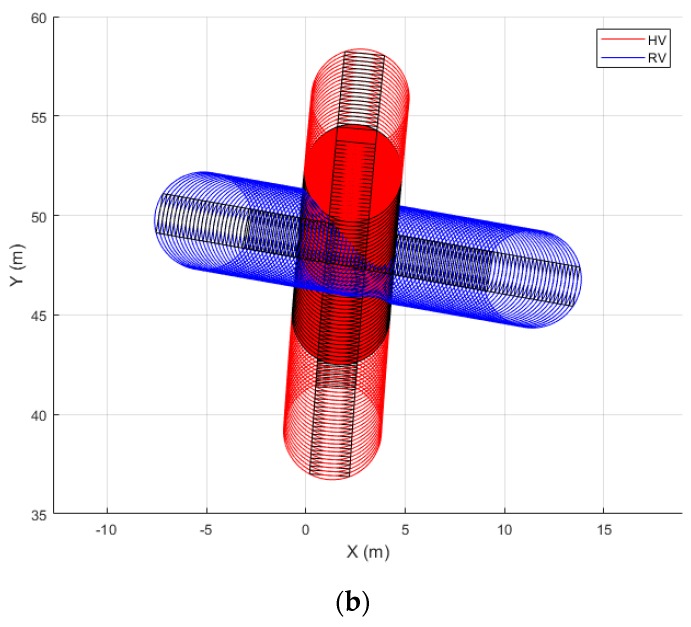
Collision event detection (circles filled in red) and time-to-collision (TTC) estimation using the predicted future trajectories of the host vehicle (HV) and the remote vehicle (RV). (**a**) Preliminary risk assessment step for collision detection; (**b**) detailed risk assessment step for TTC estimation.

The risk assessment process consists of two stages: the preliminary assessment and the detailed assessment. Figure 4 presents an example of collision event detection and TTC estimation through the two assessment stages. In the preliminary assessment stage, the future positions of the vehicles are computed using a coarse time step, which is computed as

(25) $$\Delta t_{coarse}=\frac{\sqrt{{\left(R_{HV}+R_{RV}\right)}^{2}+{\left(R_{HV}+R_{RV}\right)}^{2}}}{\max\left(v_{HV},v_{RV}\right)}=\frac{\sqrt{2}\left(R_{HV}+R_{RV}\right)}{\max\left(v_{HV},v_{RV}\right)}$$

where $v_{HV}$ and $v_{RV}$ are the speed of the host vehicle and the remote vehicle, respectively. This can be considered as a maximum time step for the preliminary risk assessment, for the collision detection algorithm can fail if longer time steps are used. When the target speed is similar to or lower than the host vehicle speed, longer $\Delta t_{coarse}$ is used for running the risk assessment for a possible collision with a large remote target such as a bus, while shorter $\Delta t_{coarse}$ is used for running the assessment for a possible collision with a small target such as a pedestrian. If a collision is detected in the preliminary stage, the future positions of the vehicles are computed using a fine time step in the detailed assessment stage, so that the TTC output is at a resolution of 0.01 s, which corresponds to a distance of a few tens of centimeters in the case of driving on a highway (about 33 cm for a vehicle traveling at 120 km/h).

If a collision is detected in the risk assessment process, an appropriate collision warning is generated to the host vehicle through the HMI according to the estimated TTC. In the case of the detection of multiple collision events, collision warning is generated for the collision associated with the shortest TTC estimation. Table 8 describes the warning generation conditions used in this work, which are similar to those of Daimler PRE-SAFE [12] and Mobileye FCW [37]. Following the suggestions made in the USDOT report on vehicular safety communications [28], we consider four collision warning stages, which include “no threat” in gray, “threat detected” in green, “inform driver” in yellow, and “warn driver” in red. In addition to visual warning, audible warning is generated for the yellow and the red warning level.

## 5. Experiments

The performance of the proposed collision warning system was evaluated experimentally in a simulation environment. Performing tests on vehicular safety systems using a driving simulator is a safer, faster, and cheaper way for system performance evaluation and validation compared with conducting driving tests with real vehicles. In this work, we utilized MATLAB/Simulink and PreScan for designing and evaluating our vehicle collision warning system in virtual driving environments. The simulation was performed in two different types of vehicle collision scenarios: a vehicle–vehicle collision scenario and a vehicle–pedestrian collision scenario.

### 5.1. Experimental Environment

#### 5.1.1. Vehicle Configuration

**Figure 5:**
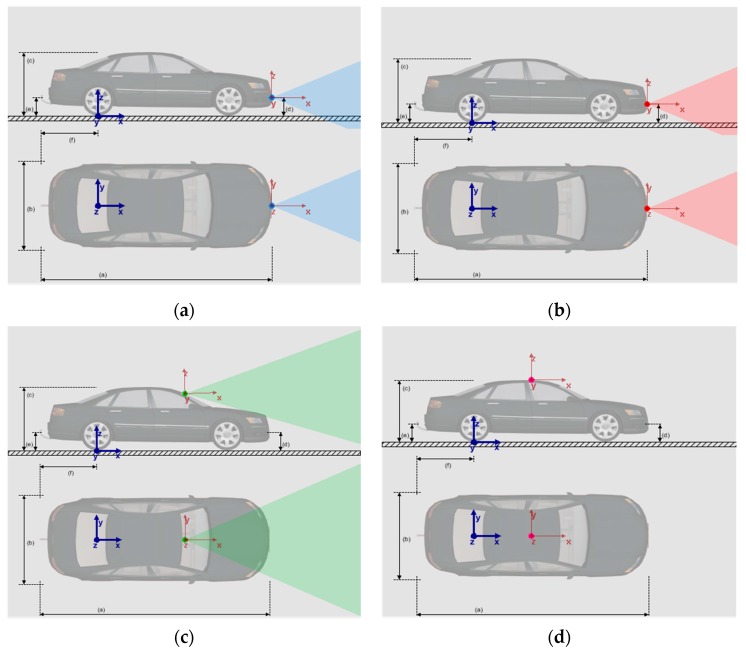

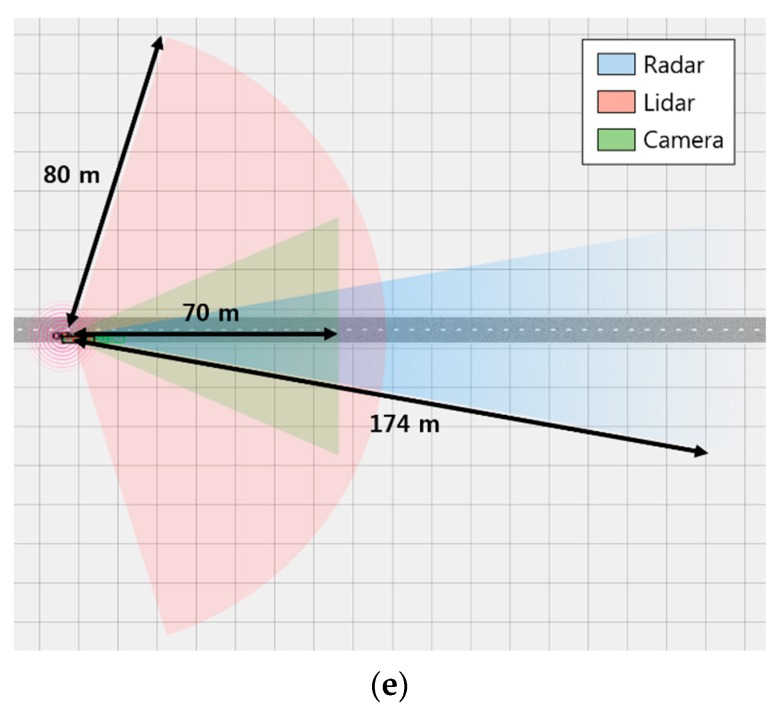
Locations of the sensors installed on the host vehicle and the sensor coverage. (**a**) Radar; (**b**) lidar; (**c**) camera; (**d**) GNSS antenna; (**e**) sensor range and FOV.

According to the specifications described in Section 3, we equipped the host vehicle with remote sensing sensors including a radar, a lidar, and a camera, as well as a DSRC transceiver for V2X communications in the simulation environment. Figure 5 shows the sensor installation illustrations and a bird’s-eye view of the vehicle setup within the PreScan model. One long-range radar and one scanning lidar were mounted on the front bumper of the vehicle, and one Mobileye camera was installed on the front windshield. For the sake of simplicity, a GNSS antenna was installed on the center of the bounding box of both the vehicles and the VRUs in our experiments such that the GNSS measurements, obtained from the host vehicle as well as from the remote targets via V2X communications, represent the center position of the two-dimensional bounding box. Some notable dimensions of the vehicle (used for both the host vehicle and the remote vehicle) shown in Figure 5a–d are as follows: length = 5.208 m; width = 2.029 m; and height = 1.447 m. The range and the FOV of each type of sensor installed on the host vehicle are presented in different colors in Figure 5e.

**Figure 6:**
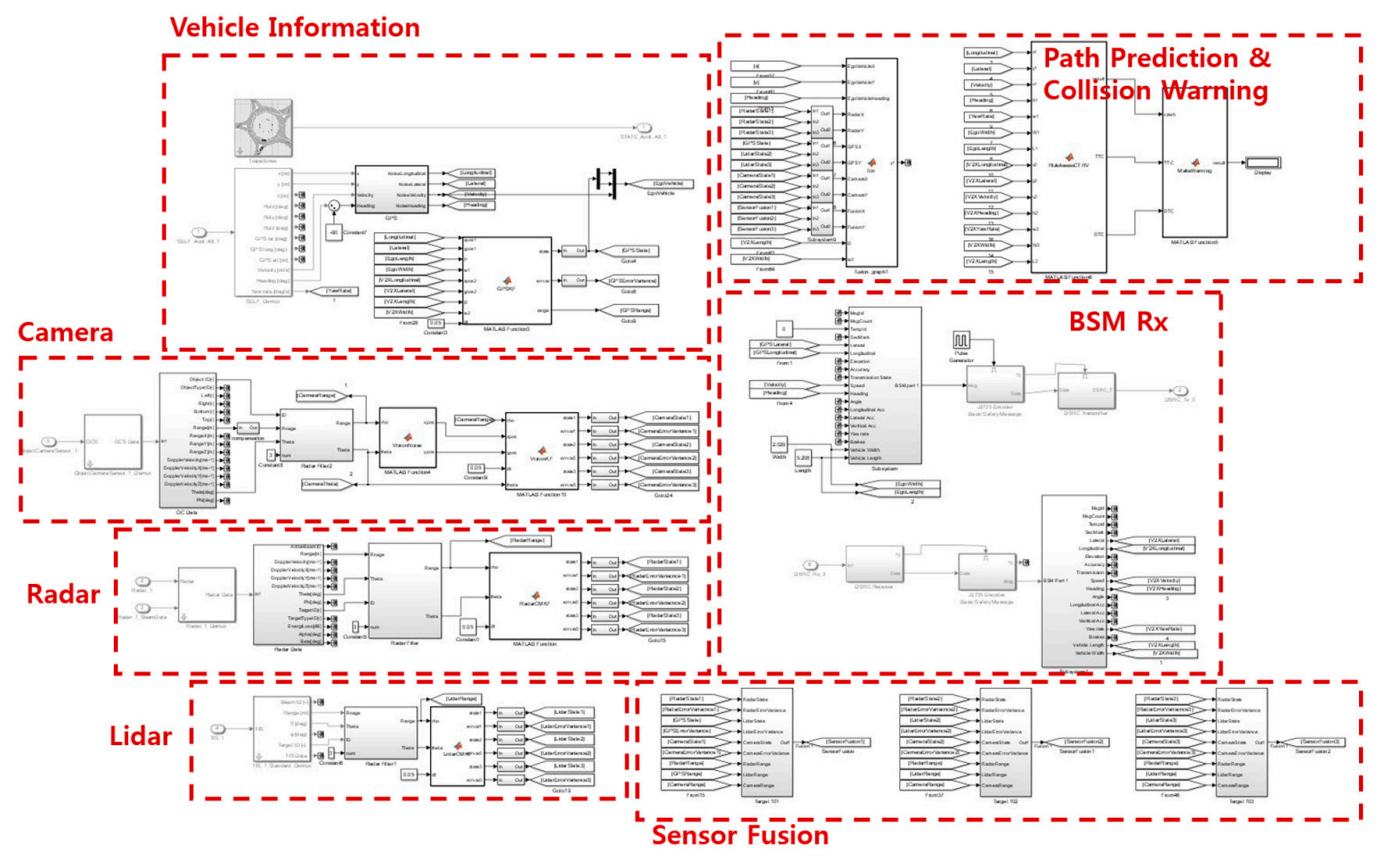
Simulink blocks and subsystems designed for the proposed vehicle collision warning system.

Figure 6 shows the Simulink blocks and subsystems constructed for the proposed vehicle collision warning system. At each time step, measurements from radar, lidar, and camera, as well as safety messages generated from remote targets were collected from the PreScan simulation environment and processed as explained in the previous section in order to estimate the target trajectory and provide the driver with an appropriate warning when a potential collision is detected.

#### 5.1.2. Vehicle–Vehicle Collision Scenario

The vehicle–vehicle collision simulation environment considered in this work is a straight-crossing-paths (SCP) scenario. The SCP scenario at non-signalized junctions ranked the highest among all crashes involving two vehicles in terms of functional years lost [38]. Furthermore, compared with other crossing path collision scenarios at intersections, the SCP scenario is the most frequent collision type when combining the number of crashes at intersections controlled with traffic light signals and stop signs as well as the intersections with no control [39].

**Figure 7:**
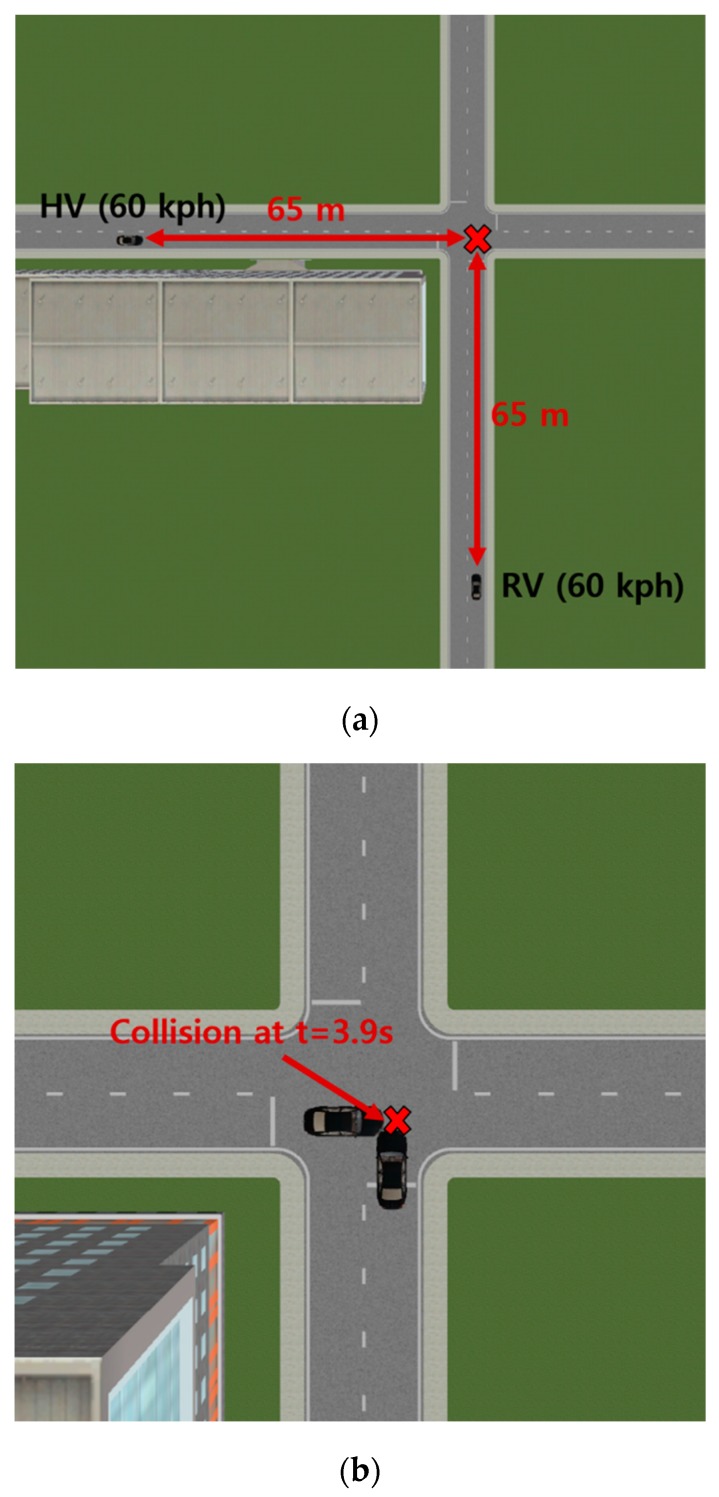
The simulation environment for the vehicle–vehicle collision scenario. (**a**) Experiment setup at the start of the simulation; (**b**) collision between the host and the remote vehicle at the end of the simulation.

A simulation environment for the SCP scenario including two vehicles—a host vehicle and a remote vehicle—was built using PreScan, as shown in Figure 7, to evaluate the performance of the proposed vehicle collision warning system in urban environments. In order to test the proposed system in a challenging yet frequently-occurring scenario, the traveling speed for both vehicles was set to 60 km/h, which corresponds to an upper boundary of average vehicle speed on urban roads with low junction density [40]. The host vehicle traveled from west to east, whereas the remote vehicle traveled from south to north. The two vehicles collided at the end of the simulation where t=3.9 s. An office building was placed in the southwest corner of the intersection to simulate perception in urban driving environments. The width of the sidewalk was set to 1.5 m, and the building was placed 3 m away from the road.

#### 5.1.3. Vehicle–Pedestrian Collision Scenario

The vehicle–pedestrian collision simulation environment considered in this work is a scenario where a pedestrian is crossing the road while a vehicle is approaching. According to the USDOT report on vehicle–pedestrian crashes [41], the top four vehicle–pedestrian pre-crash scenarios ranked based on the functional years lost are the following:

1. Pedestrian crossing the road while vehicle going straight.
2. Pedestrian crossing the road while vehicle turning right.
3. Pedestrian crossing the road while vehicle turning left.
4. Pedestrian traveling along/against traffic while vehicle going straight.

Among these four, the first scenario, which is considered for the vehicle–pedestrian collision simulation in this paper, is the most frequent vehicle–pedestrian collision type and accounts for 85 percent of functional years lost for all vehicle–pedestrian pre-crash scenarios.

**Figure 8:**
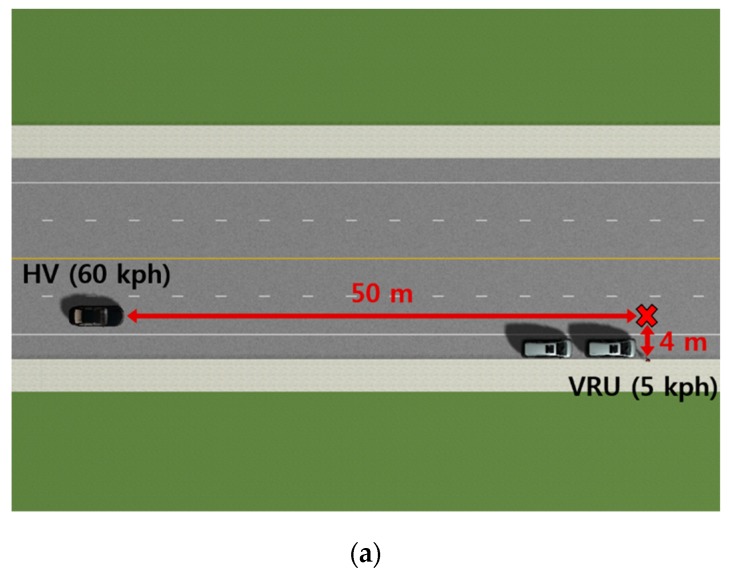

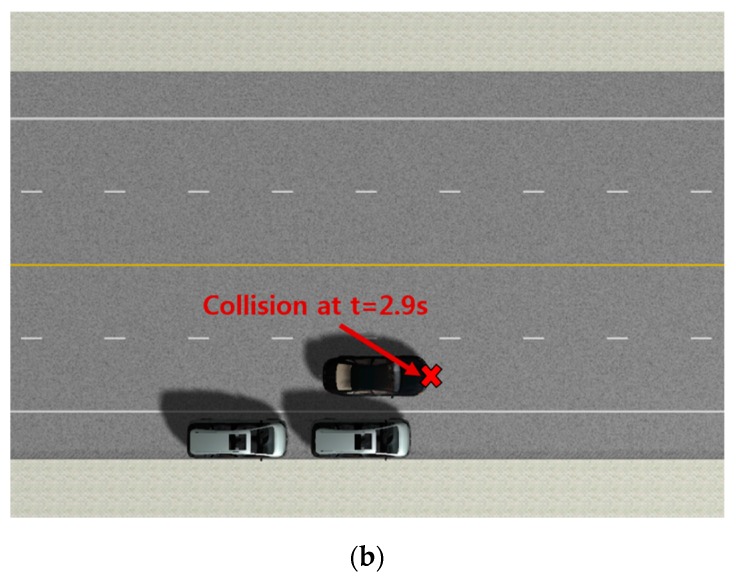
Simulation environment for the vehicle–pedestrian collision scenario: (**a**) Experiment setup at the start of the simulation; (**b**) collision between the host vehicle and the pedestrian at the end of the simulation.

A simulation environment for this vehicle–pedestrian collision scenario was designed with PreScan in conformity with the Car-to-Pedestrian Nearside Child (CPNC-50) scenario as defined in the Euro NCAP test protocol for AEB VRU systems [36]. As illustrated in Figure 8, the CPNC-50 is a collision where the center of the front side of a vehicle (i.e., 50 percent of the vehicle width) traveling straight strikes a child pedestrian who appears from the nearside, behind obstruction vehicles, and crosses the road. The test protocol also specifies that the vehicle speed should be 20–60 km/h and the pedestrian speed should be 5 km/h. In order to test the performance of the proposed system in the most challenging case, the traveling speeds for the host vehicle and the pedestrian were set to 60 km/h and 5 km/h, respectively. The host vehicle traveled from west to east, while the pedestrian traveled from south to north. At the end of the simulation, the host vehicle and the pedestrian collided at t=2.9 s. The two cars parked roadside were separated by 1 m, and their left side was positioned 1 m away along the lateral direction from the right side of the host vehicle.

### 5.2. Performance Evaluation and Analysis

#### 5.2.1. Vehicle–Vehicle Collision Scenario

**Figure 9:**
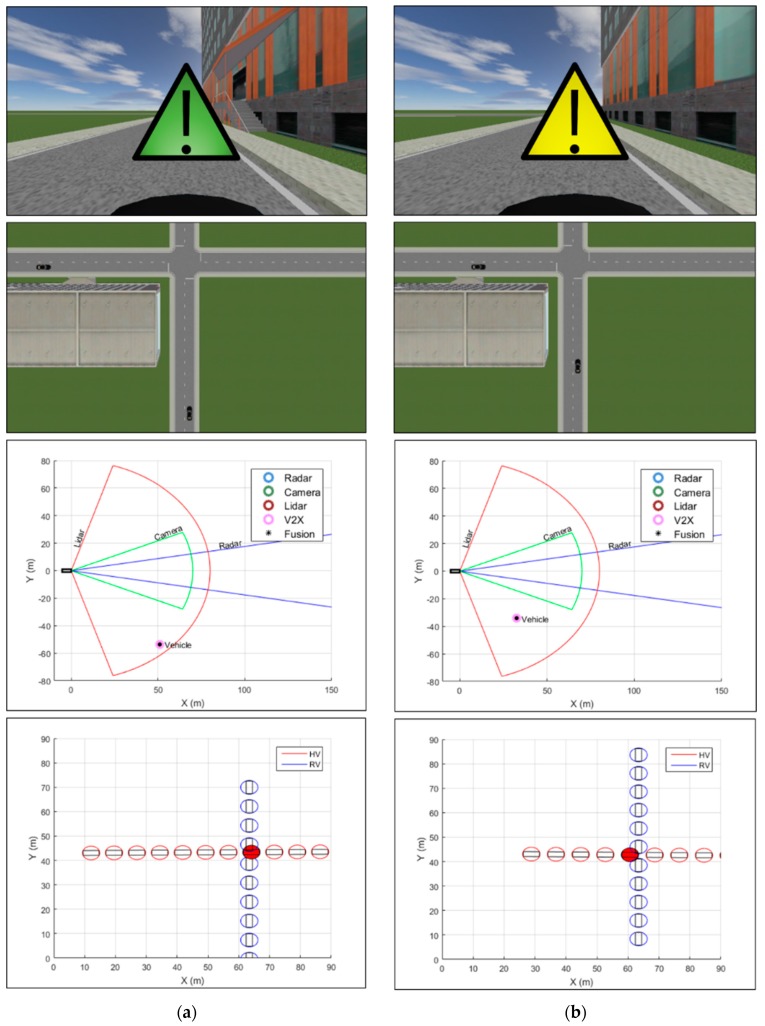

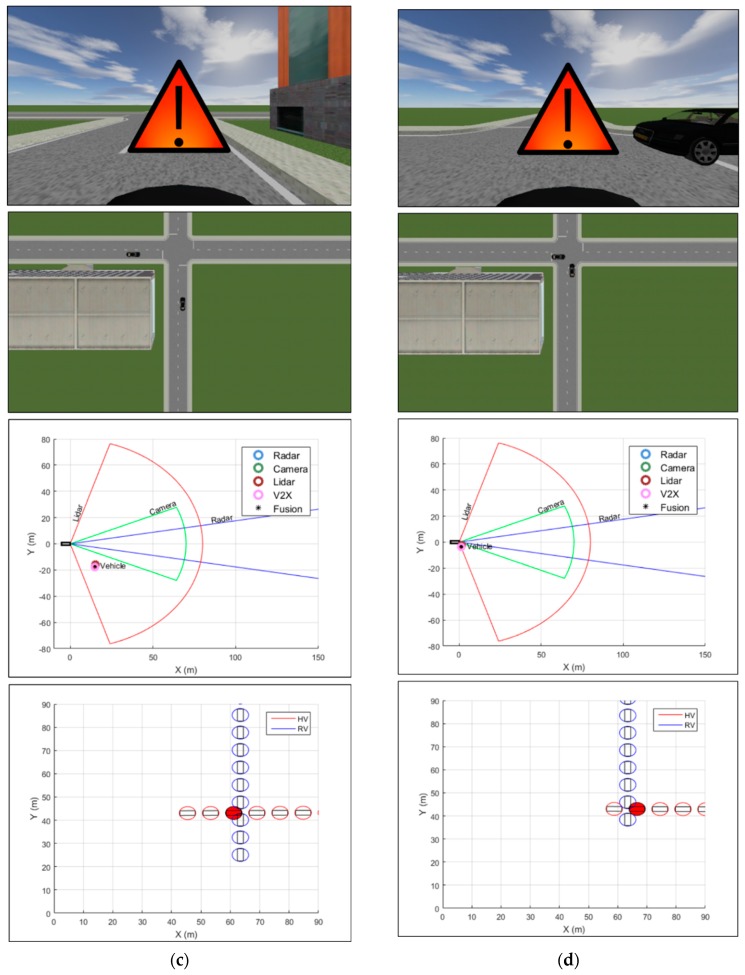
Vehicle–vehicle collision simulation results and snapshots of the experimental environment at different time points. Shown in the center of the forward-looking view image is the visual collision warning generated to the host vehicle. The bird’s-eye-view image of the road scene shows the locations of the vehicles at the corresponding time instance. The sensor fusion image shows the filtered measurements from various sensors as well as the fusion result. Trajectory prediction and risk assessment enable detection of potential collision location, which is represented by a circle colored in red. (**a**) Results for t=1 s; (**b**) results for t=2 s; (**c**) results for t=3 s; (**d**) results for the time point just before the collision.

The simulation results from the vehicle–vehicle collision scenario along with snapshots of the experimental environment at four different time instances are presented in Figure 9. A set of images shown for each simulation time point includes the forward-looking view from the perspective of the host vehicle, the top-view of the road scene, the sensor fusion result along with filtered measurements from different sources, and finally the collision detection result from the trajectory prediction and preliminary risk assessment algorithms. In the center of the forward-looking view images, an appropriate visual collision warning to the host vehicle is shown as a result of potential collision detection. The color of the visual warning represents the corresponding warning level as explained in Table 8. Throughout the simulation time, the proposed system performed well in providing proper collision warning to the host vehicle. Figure 9a,b correspond to the results for t=1 s and t=2 s, respectively, where, despite the lack of on-board sensor measurements, the results demonstrate successful collision warning based on the BSM data obtained through V2V communications. After t=3 s, the line of sight to the remote vehicle was no longer blocked by the building near the intersection and thus collision detection was carried out with measurements from the lidar in addition to the BSM, as shown in Figure 9c,d.

**Figure 10:**
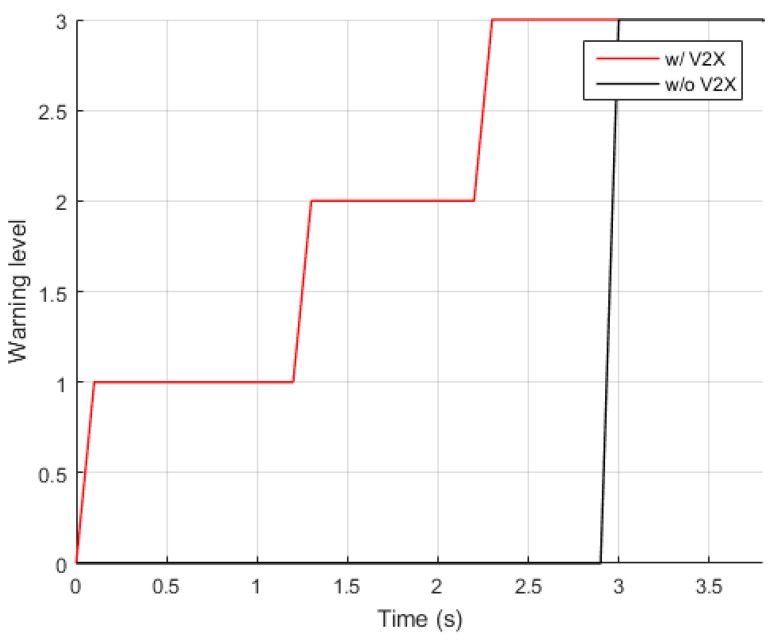
Collision warning generated over time in the vehicle–vehicle collision scenario.

Figure 10 illustrates the level of the collision warning generated throughout the simulation period from one sequence of the vehicle–vehicle collision simulation. In order to investigate the effectiveness of the implementation of vehicular communications in the SCP collision scenario considered in this paper, the collision warning results provided by the proposed system and those by the identical system with vehicular communications turned off were compared. The proposed system successfully detected a potential collision at the start of the simulation and generated a level-1 warning at t=0.1 s. A level-2 warning and a level-3 warning were subsequently provided to the host vehicle at t=1.3 s and t=2.3 s, respectively, which would give the driver sufficient time to react and slow down the vehicle speed. On the other hand, without vehicular communications the collision warning system failed to provide any warning until only 0.9 s before the collision, which is insufficient for a driver to avoid or mitigate the collision, considering the typical human reaction time of 1.5 s to apply brakes upon the occurrence of unexpected events [42].

In order to analyze the simulation result in a quantitative manner, we collected the TTC estimates from 10 separate experiments of the vehicle–vehicle collision scenario and grouped them into 1-s bins as presented in Table 9. The mean and the standard deviation of the error in the TTC estimates were computed for each bin. In this analysis, we observe that the accuracy of the TTC estimates becomes significantly better as the actual TTC becomes smaller. The average error and the standard deviation in the TTC estimates for ${\mathrm{TTC}}_{Actual}\leq 1$ are smaller than those for $3<{\mathrm{TTC}}_{Actual}\leq 4$ by a factor of 20 and 5, respectively. The results confirm that the proposed system is well capable of providing the driver with accurate warning messages in the vehicle–vehicle collision scenario considered in this work.

#### 5.2.2. Vehicle–Pedestrian Collision Scenario

**Figure 11:**
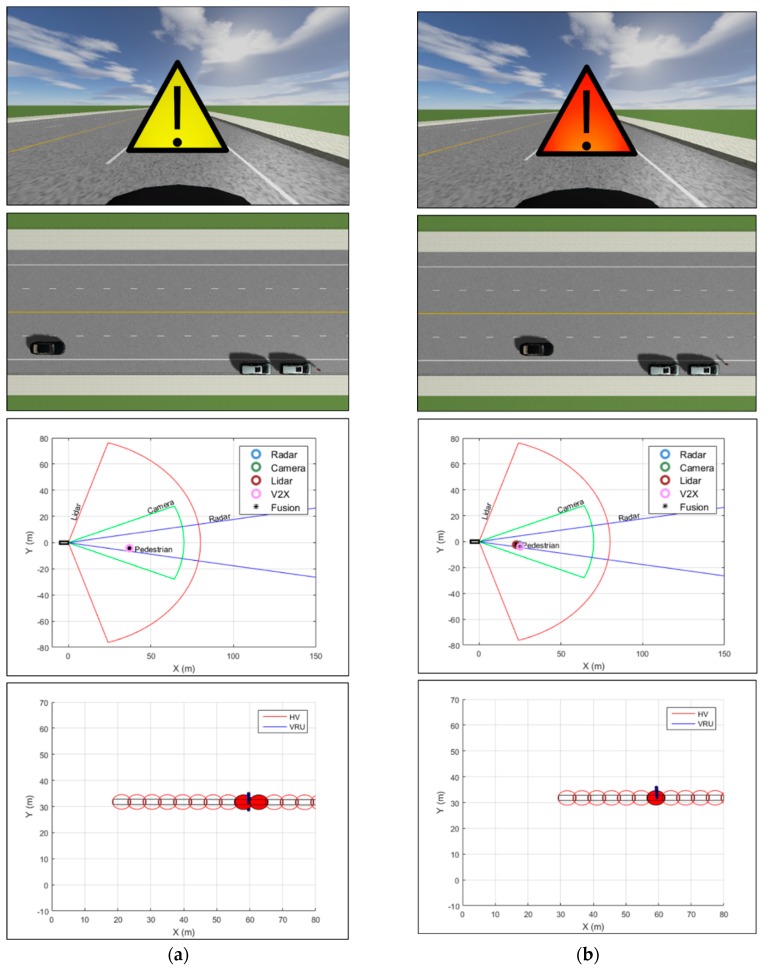

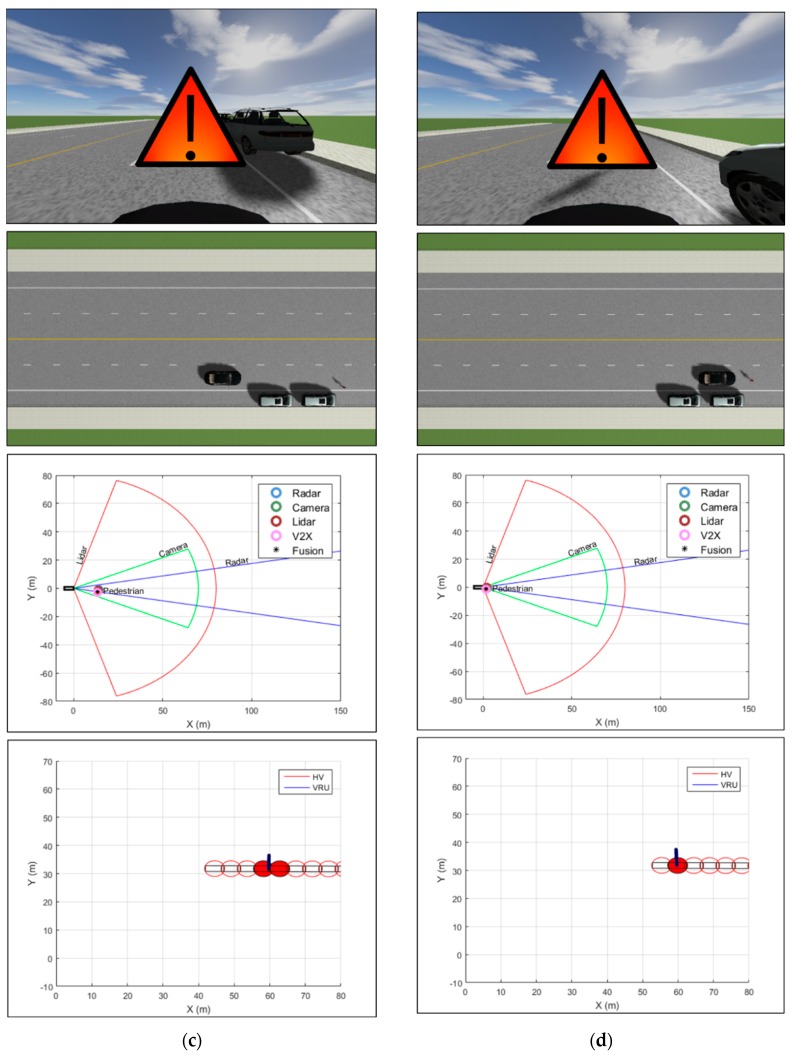
Vehicle–pedestrian collision simulation results and snapshots of the experimental environment at different time points. Shown in the center of the forward-looking view image is the visual collision warning generated to the host vehicle. The bird’s-eye-view image of the road scene shows the locations of the host vehicle and the pedestrian at the corresponding time instance. The sensor fusion image shows the filtered measurements from various sensors as well as the fusion result. Trajectory prediction and risk assessment enable detection of potential collision location, which is represented by a circle colored in red. (**a**) Results for t=0.7 s; (**b**) results for t=1.4 s; (**c**) results for t=2.1 s; (**d**) results for the time point just before the collision.

The simulation results from the vehicle–pedestrian collision scenario along with snapshots of the experimental environment at four different time instances are presented in Figure 11. Four sets of images are presented for four different time instances. For each corresponding time point, the forward-looking view from the perspective of the host vehicle shows the visual collision warning given to the host vehicle, whereas the bird’s-eye-view image of the road scene displays where the host vehicle and the pedestrian are located. In the sensor fusion images, we present the positioning results at the corresponding time as well as the results obtained with each sensor. Finally, the collision detection results from the trajectory prediction and preliminary risk assessment algorithms are shown in the images on the bottom. The different colors of the visual warning indicate different warning levels, which are previously defined in Table 8. Throughout the simulation time, we observe that the proposed collision warning system successfully generated appropriate warnings to the host vehicle. Figure 11a corresponds to the results for t=0.7 s, where potential collision with the pedestrian is detected solely based on the PSM data obtained with V2P communications. After the simulation time reached t=1.4 s, the line of sight to the pedestrian was no longer blocked by the two cars parked roadside and thus the collision detection results were based on the measurements collected from the radar, the lidar, the camera, and the PSM collected from the pedestrian, as shown in Figure 11b–d.

**Figure 12:**
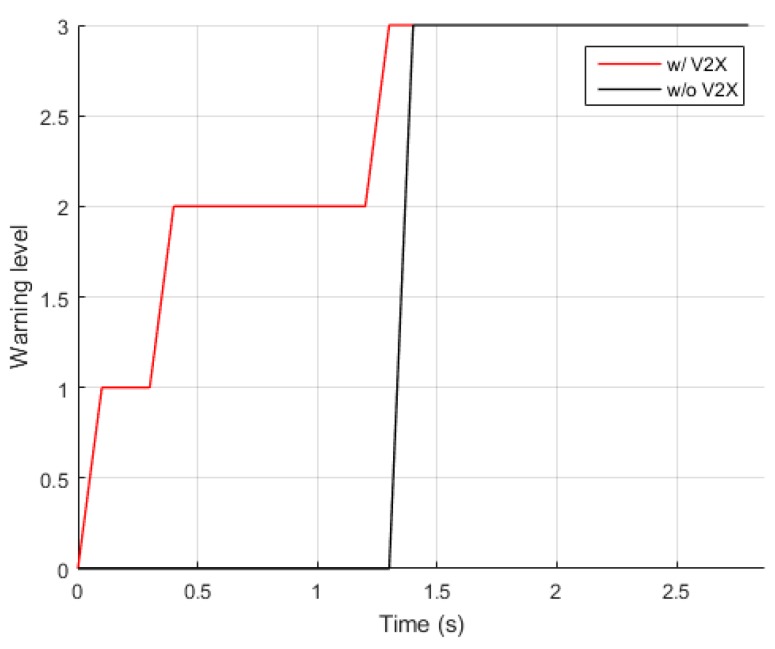
Collision warning generated over time in the vehicle–pedestrian collision scenario.

The different levels of the collision warning generated from a single sequence of the vehicle–pedestrian collision simulation are shown in Figure 12. The collision warning results provided by the proposed system and those by the identical system with vehicular communications turned off were plotted together to compare the performance of the two systems in the vehicle–pedestrian collision scenario we considered in this work. A potential collision was successfully detected with the proposed system at the start of the simulation and generated a level-1 warning at t=0.1 s. The level of collision warning was soon raised to level 2 at t=0.4 s, which corresponds to 2.5 s before the collision. Although in this particular sequence the level-2 warning was activated 0.1 s later than it was expected, a warning offset of 0.1 s is entirely acceptable in the case of the vehicle–pedestrian scenario we previously defined, considering that the remaining time before the collision is longer than 2 s. A level-3 collision warning was correctly generated to the host vehicle 1.6 s prior to the collision. In the case of the collision warning system without vehicular communications, a warning was not generated until 1.5 s before the collision because the line of sight to the pedestrian had been occluded by the cars parked on the side of the road. When taking into account the typical reaction time of 1.5 s to apply brakes in case of unexpected events [42], this warning may appear to give an attentive driver just enough time to react and slow down; however, it would still be difficult to avoid the collision when considering the vehicle braking distance.

Table 10 presents the errors in the TTC estimates collected from 10 individual sequences of the vehicle–pedestrian collision simulation. We grouped the TTC estimates into 1-s bins in order to quantitatively investigate how the performance of the proposed system depends on the actual time remaining before the collision. For each 1-s bin, we computed the mean and the standard deviation of the error in the TTC estimates. The results clearly show that the accuracy of the TTC estimates becomes significantly higher as the vehicle nears the collision location. The average error and the standard deviation of the TTC estimates for ${\mathrm{TTC}}_{Actual}\leq 1$ are smaller than those for $2<{\mathrm{TTC}}_{Actual}\leq 3$ by a factor of 10 and 4, respectively, which shows similar improvement compared to the two sample groups from the results of the vehicle–vehicle collision simulation. The analysis confirms that the proposed system successfully generates timely warnings to the host vehicle in the vehicle–pedestrian collision scenario considered in this paper.

## 6. Conclusions

In this paper, we present the development of a vehicle collision warning system based on multisensors and V2X communications. On-board sensors including radar, lidar, and camera systems that have already been adopted in production vehicles are chosen for this work such that by adding V2X communication devices to the vehicle, we can evaluate the benefits of introducing V2X communications to today’s vehicles in terms of road safety. The proposed design employs a Kalman filter-based approach for high-level fusion of V2X communications and on-board automotive sensors for remote sensing. Based on the TTC estimate result from the trajectory prediction and the risk assessment steps, an appropriate visual and audible warning is provided to the driver prior to the collision. The performance of the proposed system is evaluated in virtual driving environments, where two types of vehicle collision scenarios are considered: a vehicle–vehicle collision in an SCP scenario and a vehicle–pedestrian collision in the Euro NCAP test scenario. The results from the proof-of-concept test demonstrate that the proposed system enables higher driver and pedestrian safety through improved perception performance and proper collision warning, even in situations where collision mitigation is difficult with existing safety systems. For future work, we plan to implement the proposed vehicle collision warning method in an in-vehicle prototyping system and evaluate the performance in various driving conditions. In order to ensure the collision warning application reliability, we also aim to investigate the effects of various factors (e.g., distance between vehicles and transmission power) that could adversely affect the reliability of V2X communications.

## Figures and Tables

**Table 1:** Automotive radar specifications.

Type	Delphi ESR	Bosch LRR3	Continental ARS 30X
Frequency band	76.5 GHz	76–77 GHz	76–77 GHz
Range	174 m	250 m	200 m
Range accuracy	0.5 m	0.1 m	0.25 m
Angular accuracy	0.5 deg	n/a ^1^	0.1 deg
Horizontal FOV	20 deg	30 deg	17 deg
Data update	50 ms	80 ms	66 ms

^1^ Information not provided in the specification.

**Table 2:** Automotive lidar specifications.

Type	Ibeo Scala B3.0
Laser wavelength	905 nm
Range	80 m
Range accuracy	0.1 m
Horizontal resolution	0.25 deg
Horizontal FOV	145 deg
Data update	40 ms

**Table 3:** Automotive vision sensor specifications.

Type	Mobileye Camera
Frame size	640 × 480 pixels
Range	70 m (detection)
	100 m (tracking)
Accuracy	5% error at 45 m
	10% error at 90 m
Horizontal FOV	47 deg

**Table 4:** Vehicular wireless communications characteristics.

Type	WAVE Standards
Frequency	5.850–5.925 GHz
Channel	1 CCH, 6 SCH
Bandwidth	10 MHz
Data rate	3–27 Mbps
Maximum range	1000 m

**Table 5:** Basic safety message (BSM) format.

Message	Content
BSM Part I	Message count
	Temporary ID
	Time
	Position (latitude, longitude, elevation)
	Position accuracy
	Transmission state
	Speed
	Heading
	Steering wheel angle
	Acceleration
	Yaw rate
	Brake system status
	Vehicle size (width, length)
BSM Part II	Event flags
	Path history
	Path prediction
	RTCM package

**Table 6:** Personal safety message (PSM) format.

Message	Content
PSM	Personal device user type
	Time
	Message count
	Temporary ID
	Position (latitude, longitude, elevation)
	Position accuracy
	Speed
	Heading

**Table 7:** Information accuracy for the BSM and the PSM.

Message	Type	Accuracy
BSM	Position	0.5 m
	Heading	0.3 deg
	Speed	0.3 m/s
	Yaw rate	0.5 deg/s
PSM	Position	1.5 m
	Heading	5 deg
	Speed	0.56 m/s

**Table 8:** Conditions for the vehicle collision warning stages.

Condition	Stage	Warning Type	Color
No collision detected	No threat (Level 0)	Visual	Gray
TTC>2.6	Threat detected (Level 1)	Visual	Green
1.6<TTC≤2.6	Inform driver (Level 2)	Visual and audible	Yellow
TTC≤1.6	Warn driver (Level 3)	Visual and audible	Red

**Table 9:** Errors in the estimated TTC for the vehicle–vehicle collision scenario.

Data Range	Mean (s)	SD (s)
$3<{\mathrm{TTC}}_{Actual}\leq 4$	0.08	0.05
$2<{\mathrm{TTC}}_{Actual}\leq 3$	0.05	0.05
$1<{\mathrm{TTC}}_{Actual}\leq 2$	0.03	0.02
${\mathrm{TTC}}_{Actual}\leq 1$	0.004	0.01

**Table 10:** Errors in the estimated TTC for the vehicle–pedestrian collision scenario.

Data Range	Mean (s)	SD (s)
$2<{\mathrm{TTC}}_{Actual}\leq 3$	0.01	0.04
$1<{\mathrm{TTC}}_{Actual}\leq 2$	0.007	0.03
${\mathrm{TTC}}_{Actual}\leq 1$	0.001	0.01

## Abbreviations

ADAS	Advanced Driver Assistance Systems
AEB	Automatic Emergency Braking
BSM	Basic Safety Message
CTRV	Constant Turn Rate and Velocity
DSRC	Dedicated Short-Range Communications
FOV	Field of View
FCW	Forward Collision Warning
GNSS	Global Navigation Satellite System
HMI	Human-Machine Interface
NCAP	New Car Assessment Program
NLOS	Non-Line of Sight
OBU	On-Board Unit
PSM	Personal Safety Message
SCP	Straight Crossing Paths
TTC	Time-to-Collision
V2X	Vehicle-to-Everything
V2I	Vehicle-to-Infrastructure
V2P	Vehicle-to-Pedestrian
V2V	Vehicle-to-Vehicle
VRU	Vulnerable Road User
WAVE	Wireless Access in Vehicular Environments
